# The Effects of Acute Exposure to Ammonia on Oxidative Stress, Hematological Parameters, Flesh Quality, and Gill Morphological Changes of the Large Yellow Croaker (*Larimichthys crocea*)

**DOI:** 10.3390/ani13152534

**Published:** 2023-08-06

**Authors:** Meijie Guo, Zhenkun Xu, Hongzhi Zhang, Jun Mei, Jing Xie

**Affiliations:** 1 College of Food Science and Technology, Shanghai Ocean University, Shanghai 201306, China; m210311056@st.shou.edu.cn (M.G.); m200300892@st.shou.edu.cn (Z.X.); m200300888@st.shou.edu.cn (H.Z.); 2 National Experimental Teaching Demonstration Center for Food Science and Engineering, Shanghai Ocean University, Shanghai 201306, China; 3 Shanghai Engineering Research Center of Aquatic Product Processing and Preservation, Shanghai 201306, China

**Keywords:** ammonia stress, antioxidant enzymes, serum components, gill tissue injury

## Abstract

**Simple Summary:**

The large yellow croaker is one of the most economically valuable marine fish in China. However, ammonia accumulates in its blood through the gills and causes fish poisoning. In the past years, few studies have focused on the toxic effects of acute ammonia exposure on fish, especially on the large yellow croaker. Therefore, the aim of this study was to evaluate the effects of acute ammonia exposure on the hematological parameters, hepatic oxidative stress, gill morphological changes and meat quality of the large yellow croaker. Exposure of large yellow croakers to four ammonia solutions at concentrations of 0, 2.96, 5.92, and 8.87 mg/L for 48 h revealed changes in serum composition, as well as oxidative stress, tissue damage in liver and gills, accumulation of free amino acids, and loss of nucleotides.

**Abstract:**

Ammonia is considered to be the major chemical pollutant causing fish poisoning in aquaculture. This research aimed to evaluate the impact of acute ammonia exposure on the large yellow croaker’s meat quality, gill morphology, liver oxidative stress, and hematological parameters. The fish were exposed to total ammonia nitrogen concentrations of 0, 2.96, 5.92, and 8.87 mg/L for 48 h, respectively. The findings demonstrated that all ammonia-exposed fish had higher liver lactate dehydrogenase and glutamic oxalate transaminase activities. The glucose, blood urea nitrogen, and creatinine levels in 8.87 mg/L total ammonia nitrogen (TAN) were higher than other samples. The total protein, albumin, and triglyceride levels in serum decreased significantly in ammonia-exposed samples. After 48 h of ammonia exposure, superoxide dismutase activities showed a 76.1%, 118.0%, and 156.8% increase when fish were exposed to 2.96, 5.92, and 8.87 mg/L TAN, respectively. Catalase activities and glutathione contents were considerably higher (*p* < 0.05) in all ammonia-treated samples compared to 0 mg/L TAN. The ammonia-treated gill lamellae become thicker, shorter, and curved. Additionally, the ammonia exposure resulted in the accumulation of free amino acids and the loss of nucleotides. The inosine monophosphate and adenosine monophosphate contents in the flesh were decreased after 12 h of exposure to 2.96, 5.92, and 8.87 mg/L ammonia compared to the control group. Overall, large yellow croakers exposed to ammonia for 6 h presented not only changes in serum composition but also oxidative stress, liver and gill tissue damage and flesh quality deterioration.

## 1. Introduction

Ammonia is a ubiquitous contaminant in aquatic environments and is a byproduct of protein catabolism and metabolism [1]. Different studies have found that elevated ammonia levels lead to a range of deleterious effects such as abnormal enzyme metabolism, organ damage, oxidative stress, neurotoxicity, and immunosuppression [2]. Fish are more susceptible to damage from oxidative stress due to the complex aquatic environment. Oxidative stress has been shown to occur when fish are stressed by harmful factors such as nitrite [3], cadmium [4], bisphenol A [5], polypropylene microplastics [6], and pesticides [5]. Especially in high-ammonia environments, aquatic animals may suffer from oxidative stress due to reactive oxygen species (ROS) production. Oxidative stress is mainly caused by the excessive accumulation of free radicals and disruption of antioxidant enzyme systems. Ammonia-induced oxidative stress disrupts the oxidant–antioxidant balance by producing excessive ROS. Fortunately, fish can alter the activities of antioxidant enzymes and nonenzymatic antioxidants (glutathione) to scavenge ROS and balance the ratio between oxidants and antioxidants, thereby preventing oxidative damage [7]. Previous studies have shown that ammonia exposure can alter glutamate transaminase, glucose, and glutamate oxaloacetate transaminase concentrations in fish, and that the extent of the changes is dependent on the duration of the stress and the ammonia content [8]. In contrast, ROS overproduction and oxidative stress are the main causes of acute liver lesions, which may in turn continue to compromise the antioxidant defense system. In addition, gills are the main area for gas exchange and waste excretion in fish. According to studies, fish can change the morphology of their gills in response to stressful environments [9]. For example, Miron et al. [10] reported that exposure to ammonia in water resulted in morphological changes in the gills of silver catfish, including plate fusion of epithelial cells and edema. However, there are not many studies on the morphological changes in gills of large yellow croakers under ammonia stress.

Fish quality, including nutritional and flavor components, has been reported to be mainly influenced by dietary components or environmental factors [11]. Free amino acids and nucleotides are important indicators of fish meat quality and freshness [12]. Lv et al. [13] found that hypoxia reduces protein content in *Oreochromis niloticus* and may impair fish quality by reducing flavor-related amino acids (sum of flavor amino acids and sum of delicious amino acids). However, *Acipenser schrenckiizai* showed no significant changes in meat quality (e.g., total protein and crude fat) after anhydrous transportation at 4 °C [14]. Lipid metabolism is the key to adaptation to ammonia exposure. Carneiro et al. observed significant reductions in total cholesterol, triglyceride, and lactate dehydrogenase levels in *Piaractus mesopotamicus* after ammonia exposure, suggesting that lipids are catabolized to promote better energy supply through catabolic pathways in response to ammonia stress [15]. Although the effects of stress on fish quality have been reported, the effects of acute ammonia exposure on the large yellow croaker remain unclear. Studying the metabolic changes in fish is beneficial to understand the effect of ammonia nitrogen on fish quality and to explore the mechanism of fish detoxification.

In China, the large yellow croaker (*Larimichthys crocea*) is an important farmed marine fish [16,17]. The accumulation of ammonia is exacerbated by increasing feed residues formed by excessive use of protein feeds, high-density aquaculture, and low frequency of water renewal. Many studies have been conducted on the toxic effects of chronic ammonia exposure on fish. However, few studies have focused on the toxic effects of acute ammonia exposure on the large yellow croaker. Therefore, the aim of this study was to evaluate the toxic effects of ammonia exposure on large yellow croakers in terms of serum biochemical indices, physiological stress, and gill morphology induced by acute ammonia stress.

## 2. Materials and Methods

### 2.1. Experimental Fish and Experimental Procedure

The large yellow croakers (600 g ± 1 g) were obtained from Shanghai Luchao Port Market and temporarily cultured for 48 h in a 400 L tank. The parameters of the temporarily cultured water are listed as follows: the seawater temperature was kept at 20 ± 1 °C, dissolved oxygen (DO) was kept at 7.0 mg/L, and the pH and salinity of the saltwater were 7.5 and 22‰, respectively. The waterborne total ammonia nitrogen levels were determined using the technique of Molayemraftar et al. [18]. The levels of un-ionized ammonia (UIA) were denoted using the general equation

(1) $${NH}_{3}=\frac{{NH}_{3}+{{NH}_{4}}^{+}}{1+10^{\left(pK_{a}-pH\right)}}.$$

The equation of Klüver et al. [19] was used to compute $pK_{a}$.

(2) $$pK_{a}=0.09018+\frac{2729.92}{T},T=Kelvin=273+T℃.$$

$pK_{a}$ is the dissociation constant and T is the Kelvin temperature.

### 2.2. Determination of LC_50_

Ammonia solutions (10 g/L) were prepared in seawater using ammonium chloride (Aladdin Biotechnology Co., Ltd., Shanghai, China). After acclimation, fish (*n* = 72) were randomly distributed into 6 separate tanks (100 L, 12 fish/tank) and exposed to 0, 1.92, 3.68, 7.07, 13.58, and 26.06 mg/L total ammonia nitrogen (TAN) for 48 h, respectively. All the fish stopped feeding during the experiment. The desired TAN levels were achieved by adding the ammonia solution (10 g/L). The mortality was documented every 12 h, and the results of the survival of large yellow croakers were documented. The 48 h semilethal concentration (48 h-LC_50_) of ammonia stress was calculated according to the method of Liu et al. [20].

### 2.3. Experiment of Acute Ammonia Exposure Design

Under the same experimental circumstances, the 48 h LC_50_ was 9.86 mg/L. The low (30% 48 h-LC_50_, 2.96 mg/L), medium (60% 48 h-LC_50_, 5.92 mg/L), high (90% 48 h-LC_50_, 8.87 mg/L), and control group (CK, 0 mg/L) levels of TAN were chosen as the subjects of this study and prepared in order to analyze the reaction of large yellow croakers to acute ammonia exposure. The samples were labeled as NH-2.96, NH-5.92, NH-8.87, and CK, respectively. This experiment’s content design was inspired by Liu et al. [6,20]. The fish (*n* = 360) were acutely exposed to four treatments, with 30 fish for each group, in triplicate. Every 6 h, the TAN levels in the tank were kept stable by adding a suitable quantity of NH_4_Cl solution or simulated seawater [21].

### 2.4. Sample Collection

At each sample period (0, 6, 12, 24, 36, 42, and 48 h), three fish were chosen at random for testing. The serum was collected and kept in a −80 °C refrigerator after centrifugation at 1300× *g* for 15 min at 4 °C [22]. Each fish’s back and abdomen were harvested for their flesh tissues and kept at 80 °C for subsequent study. The gill filament was removed immediately and soaked for 24 h.

Blood without anticoagulant was drawn from the tail vein of the large yellow croakers, stored at 4 °C for 12 h, then centrifuged at 3000× *g* for 20 min at 4 °C, collected, and stored at −80 °C until use. The gill filaments of the large yellow croakers were removed and immersed in a 2.5% glutaraldehyde solution for 24 h pending further analysis.

### 2.5. Biochemical Analysis of Liver

The liver (0.1 g) was homogenized in 0.9 mL of precooled saline (0.85%). The homogenized mixture was centrifuged at 3000× *g* for 15 min and the supernatant was collected for assay. Superoxide dismutase (A001-3-2), glutamic oxaloacetic transaminase (C010-2-1), lactate dehydrogenase (A020-1-2), catalase (A001-3-2), glutathione (A006-2-1), and glutamate pyruvic transaminase (C009-2-1) were measured according to the instructions of the Nanjing Built-up Kit (Jiancheng Bioengineering Institute, Nanjing, China). Malondialdehyde concentration was analyzed according to the method of Zhang et al. [23] and the results were expressed as nmol/g.

### 2.6. Analysis of Changes in Serum Components

After serum was removed from the −80 °C refrigerator, it was placed in a 4 °C refrigerator to thaw fully for 30 min before use. The following indices in the serum were analyzed using commercial kits (Jiancheng Bioengineering Institute, Nanjing, China) manufactured in Nanjing: concentrations of blood urea nitrogen (C013-2-1), total protein (A045-2-2), albumin (A028-2-1), triglycerides (A110-1-1), creatinine (C011-2-1), and glucose (F006-1-1).

### 2.7. Histological Analysis

The upper gill filaments of the second gill arch of the large yellow croakers were cut according to the way of Zhang et al. [23]. The fixed gill filaments were washed with 0.1 mol/L phosphate buffer (pH = 7.4) 3 times. The gill filaments were eluted with a gradient of 30, 50, 70, 80, 90, and 100% ethanol for 15 min. Then, the ethanol was replaced by isoamyl acetate and the samples were freeze-dried for 48 h. The gill filaments were coated with a conductive metal layer and observed using emission scanning electron microscopy (SU5000, Hitachi, Japan) at a 5 kV accelerating voltage.

### 2.8. Product Quality Analysis

The contents of free amino acids were determined with the method of Li et al. [24]. Chopped fish flesh (2 g) and 15% prechilled trichloroacetic acid (15 mL) were mixed and centrifuged at 10,000× *g* for 10 min. After repeated extraction and centrifugation, the combined supernatant was diluted to 25 mL, and 5 mL supernatant was immediately filtered through a 0.22 μm filter and analyzed by amino acid analyzer (Hitachi L-8800, Tokyo, Japan).

Nucleotide extracts were made using the technique suggested by Fang et al. [25]. High-performance liquid chromatography combined with a Shim-pack column (VP-ODS C18) was used to measure inosine monophosphate (IMP) and adenosine monophosphate (AMP). Five grams of fish was minced and homogenized, and the supernatant was extracted using 10% perchloric acid. The pH of the supernatant was adjusted to 6.5 with 1 M KOH, allowed to stand, and filtered. Volume was fixed to 20 mL using a volumetric flask, and a 10 μL aliquot was injected into the column. Chromatographic separation was performed at a flow rate of 1.0 mL/min using the following gradients of 0.01 MKHPO_4_ and 0.01 MK_2_HPO_4_ (eluent A) and HPLC grade methanol (eluent B). Nucleic acid-related compounds were measured at 254 nm using a UV spectrophotometer and expressed as mg/100 g dry matter (DM).

### 2.9. Statistical Analyses

Values are expressed as mean ± standard deviation (SD). Independent and interaction effects of time and ammonia concentration were analyzed by two-way ANOVA with reference to Slami et al. [26] (Table 1). If the interaction effect was significant, Duncan’s multiple range test was applied. Homogeneity of the samples was tested by Levene before performing Duncan’s test, and the significance level for all samples was *p* < 0.05. Microsoft Excel 2019 was used for data statistics and Origin 2019b for plotting graphical legends.

## 3. Results

### 3.1. Effects of Ammonia Exposure on Survival

**Figure 1:**
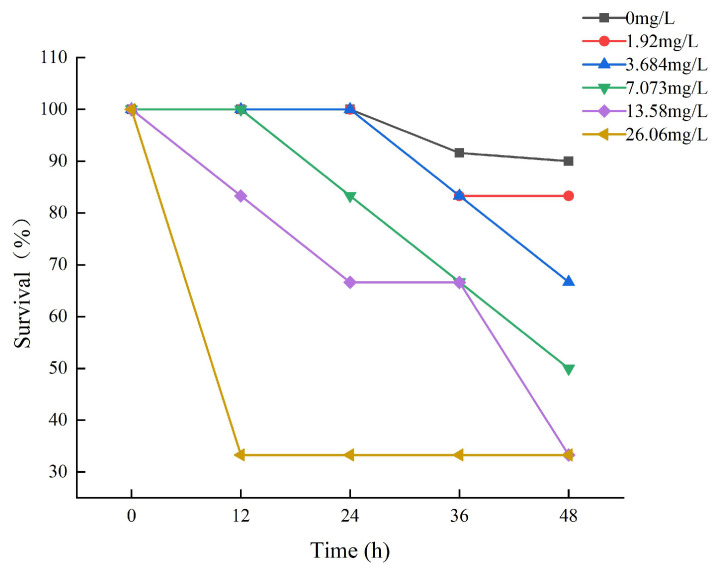
The results of the survival of large yellow croakers exposed to 0 mg/L, 1.92 mg/L, 3.684 mg/L, 7.073 mg/L, 13.58 mg/L, and 26.06 mg/L total ammonia nitrogen for 48 h, respectively.

Fish survival decreased by increasing ammonia concentrations and time of ammonia exposure during the trial (Figure 1). The survival rate of each experimental group during the exposure period is depicted in Figure 1. After 12 h of exposure, death was observed in the 13.58 mg/L and 26.06 mg/L experimental groups of large yellow croakers. The experimental group of 26.06 had a survival rate of 33.33%, which remained steady thereafter. It may be that some tenacious fish have developed a tolerance to ammonia. After 24 h of exposure, the survival rate of the 0 mg/L, 1.92 mg/L, and 3.68 mg/L experimental groups was 100%, and then it began to decline.

### 3.2. Biochemical Indices in Liver

**Figure 2:**
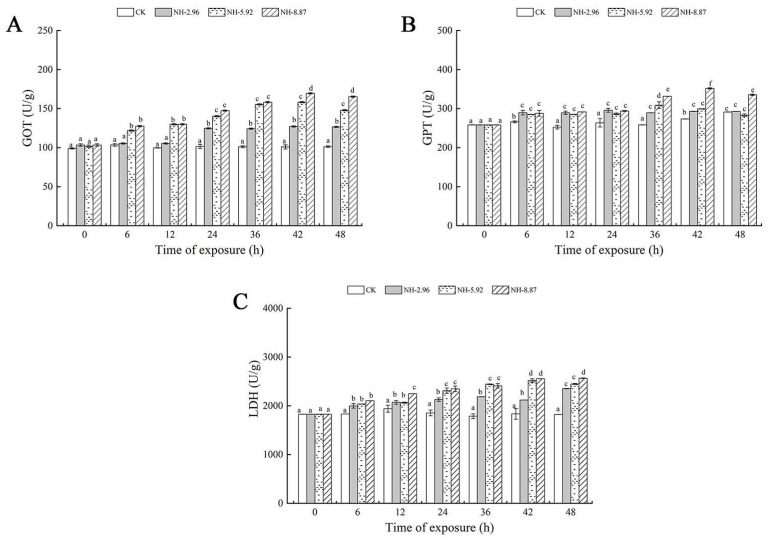
Changes in glutamic oxalate transaminase (GOT) (**A**), glutamic pyruvate transaminase (GPT) (**B**), and lactate dehydrogenase (LDH) (**C**) activities in the livers of large yellow croakers exposed to 0 mg/L (CK), 2.96 mg/L (NH-2.96), 5.92 mg/L (NH-5.92), and 8.87 mg/L (NH-8.87) total ammonia nitrogen for 48 h, respectively. Values are expressed as the mean ± S.D. Different lowercase letters indicate significant differences (*p* < 0.05) among groups. The CK nitrite group served as the control.

The results of enzyme activities related to liver injury in large yellow croakers after 48 h of ammonia exposure are presented in Figure 2. Glutamic oxaloacetic transaminase, glutamate pyruvic transaminase, and lactate dehydrogenase showed a tendency to increase and then decrease in all experimental groups exposed to ammonia. The glutamic oxaloacetic transaminase and glutamate pyruvic transaminase activities of the group labeled NH-2.96 were always higher than the other experimental groups, and the lactate dehydrogenase activity of the group labeled NH-8.87 reached its peak at 48 h, which was significantly higher than that of the other experimental groups (*p* < 0.05). This indicates that the degree of change in glutamic oxaloacetic transaminase and lactate dehydrogenase levels is closely related to the amount of ammonia exposure and exposure time.

**Figure 3:**
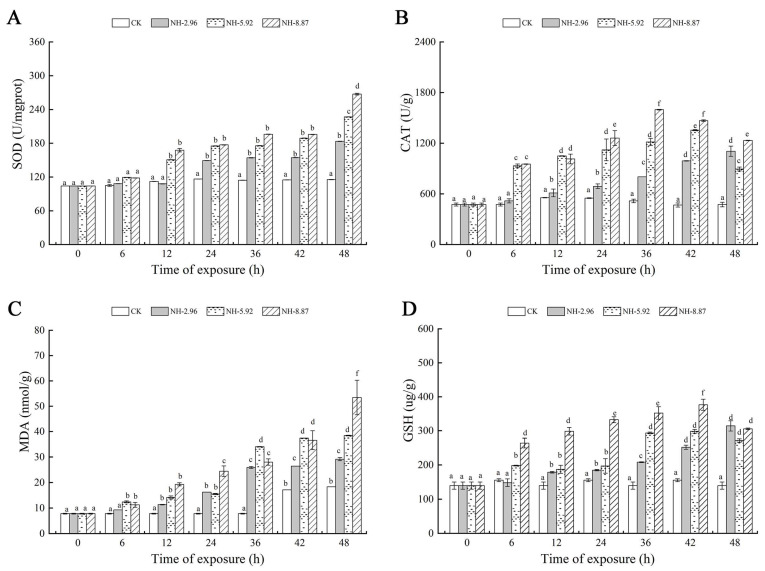
Changes in superoxide dismutase (SOD) (**A**), catalase (CAT) (**B**), glutathione (GSH) (**C**), and malondialdehyde (MDA) (**D**) levels in the livers of large yellow croakers exposed to 0 mg/L (CK), 2.96 mg/L (NH-2.96), 5.92 mg/L (NH-5.92), and 8.87 mg/L (NH-8.87) total ammonia nitrogen for 48 h, respectively. Values are expressed as the mean ± S.D. Different lowercase letters indicate significant differences (*p* < 0.05) among groups. The CK nitrite group served as the control.

Figure 3 shows the oxidative stress and oxidative damage at 48 h of ammonia exposure. The superoxide dismutase and malondialdehyde contents of the experimental groups exposed to ammonia increased with time. The superoxide dismutase and malondialdehyde contents of each experimental group peaked at 48 h, and the peaks were significantly higher than those of the CK group. The catalase and glutathione contents of the NH-5.92 and NH-8.87 experimental groups increased with the increase in exposure time, and then began to decrease. The peak of catalase activity in NH-5.92 and NH-8.87 experimental groups appeared at 42 h and 36 h, respectively. The peak of catalase activity in both NH-5.92 and NH-8.87 experimental groups appeared at 36 h.

### 3.3. Serum Composition Changes

**Figure 4:**
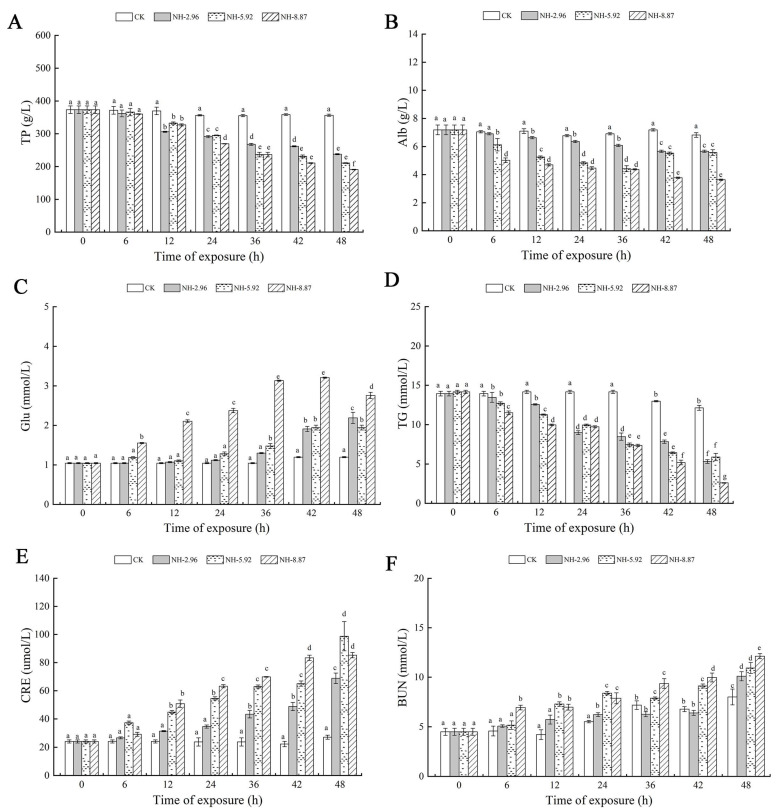
Changes in total protein (TP) (**A**), albumin (Alb) (**B**), glucose (Glu) (**C**), triglyceride (TG) (**D**), creatinine (CRE) (**E**), and blood urea nitrogen (BUN) (**F**) levels in the serum of large yellow croakers exposed to 0 mg/L (CK), 2.96 mg/L (NH-2.96), 5.92 mg/L (NH-5.92), and 8.87 mg/L (NH-8.87) total ammonia nitrogen for 48 h, respectively. Values are expressed as the mean ± S.D. Different lowercase letters indicate significant differences (*p* < 0.05) among groups. The CK nitrite group served as the control.

Figure 4 demonstrates the changes in various biochemical parameters in the serum of ammonia exposed for 48 h. The total protein and TG contents of each experimental group exposed to ammonia decreased with time. The lowest point was reached at 48 h, when the total protein and TG contents of NH-8.87 were significantly lower than those of each other experimental group (Figure 4A–D). The Alb content of all experimental groups decreased at 48 h compared to 0 h. The blood glucose content of the NH-5.92 and NH-8.87 experimental groups showed an increasing trend during the exposure period. NH-8.87 showed an increasing and then a decreasing trend, with a peak at 42 h. The triglyceride and blood urea nitrogen levels of the ammonia-exposed groups were significantly lower at 48 h than at 0 h. The triglyceride and blood urea nitrogen levels of the groups exposed to ammonia were significantly lower at 0 h than at 48 h.

### 3.4. Gill Morphology

**Figure 5:**
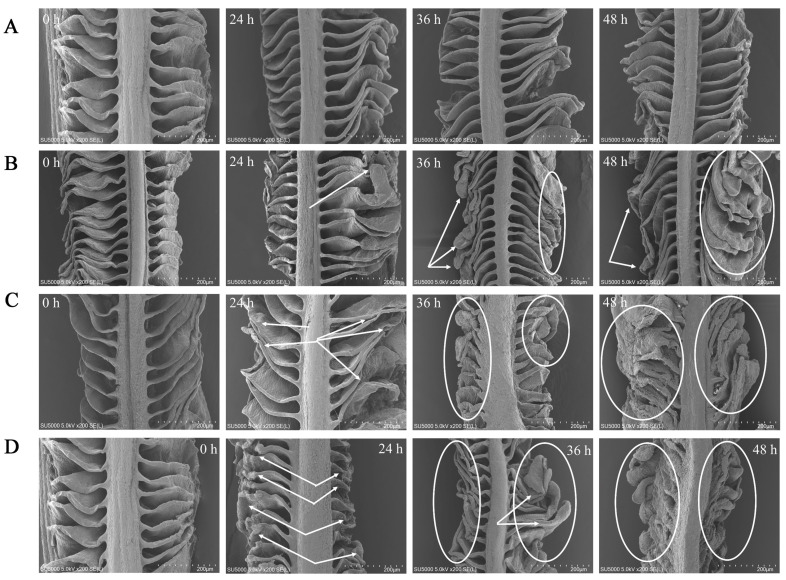
Scanning electron microscope (SEM) micrographs of the large yellow croaker gills exposed to 0 mg/L (CK, (**A**)), 2.96 mg/L (NH-2.96, (**B**)), 5.92 mg/L (NH-5.92, (**C**)), and 8.87 mg/L (NH-8.87, (**D**)) total ammonia nitrogen for 0 h, 24 h, 36 h, and 48 h. Bars = 200 µm. Whitehead arrows and circles indicated gill morphological changes.

The gill filaments were in the form of thin, flattened lamellae divided by regular interstices, and the surface structure of the gills did not undergo severe damage with the extended period of CK exposure (Figure 5A). At 24 h of exposure in NH-2.96, the gill lamellae were still separated by interlamellar gaps, but the ends of the gill lamellae at 36 and 48 h exposure became curved and slightly thicker compared to 24 h exposure (Figure 5B). The gill in NH-5.92 at 36 and 48 h exposure not only became thicker at the end of the gill lamellae but also had a reduced interlaminar space between the gill lamellae (Figure 5C). In addition, the end of the gill lamellae in NH-8.87 became thicker and thicker with the exposure time (Figure 5D). The shortest and thickest lamellae were observed at 48 h exposure in NH-8.87 samples. In addition, both filaments and lamellae in NH-5.92 and NH-8.87 samples were more curved, shorter, thicker, and more irregular than those in NH-2.96 and CK samples.

### 3.5. Body Composition Changes and Flesh Quality

**Figure 6:**
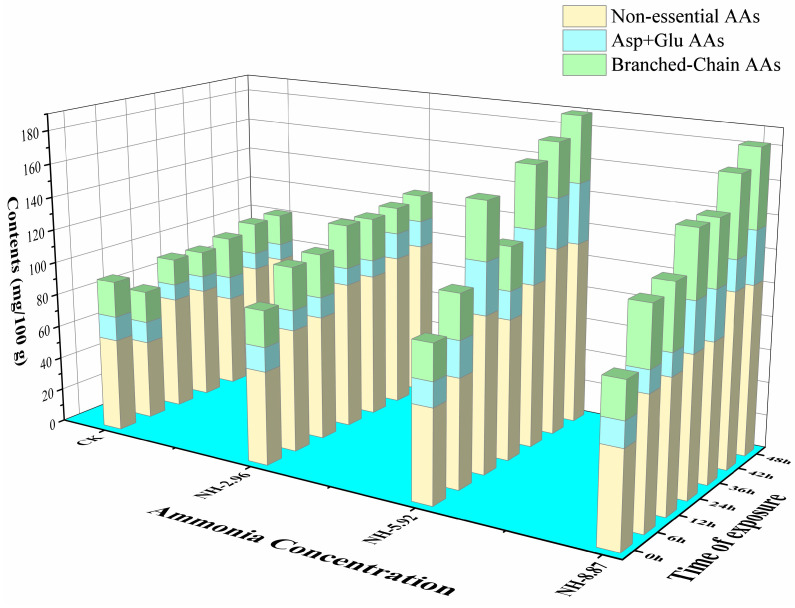
Changes in nonessential amino acids, Asp + Glu amino acids, and branched-chain amino acids in the flesh of large yellow croakers exposed to 0 mg/L (CK), 2.96 mg/L (NH-2.96), 5.92 mg/L (NH-5.92), and 8.87 mg/L (NH-8.87) total ammonia nitrogen for 48 h, respectively.

The total content of FAAs showed gradually increasing trends in ammonia-treated samples compared with CK (Table 2). The nonessential amino acid contents of fish flesh had similar trends as the total content of FAAs (Figure 6). The branched-chain amino acids in NH-5.92 and NH-8.87 were higher than CK during the ammonia exposure process.

In this study, AMP levels were clearly lower than IMP in all the samples, and the TAV content of IMP was higher than AMP. The TAV content of IMP was higher than 3, and the TAV content of AMP was lower than 1. The IMP and AMP contents of flesh in each sample showed a gradually decreasing trend with the ammonia exposure time (Table 3).

## 4. Discussion

Large yellow croakers are sensitive to ammonia and susceptible to ammonia poisoning. Fish survival will decrease as ammonia content and exposure duration increase. And continuous exposure to ammonia causes fish to accumulate ROS, which can lead to oxidative stress. To withstand stress, fish have evolved an antioxidant enzyme defense system in which antioxidant enzymes (superoxide dismutase and catalase) and antioxidants (glutathione) play critical roles in controlling ROS generation and scavenging [27]. The higher ammonia exposure content and exposure duration could activate the fish’s antioxidant defense system and then eliminate the excessive ROS produced in the body by increasing superoxide dismutase and catalase activities. The increase in the H_2_O_2_ level caused by the elevated superoxide dismutase activity may also be one of the reasons for the increase in catalase activity. The current study’s findings matched those of Zhao et al. [28], who found that antioxidant response induced by ammonia exposure led to elevated superoxide dismutase and catalase activities in the liver of *Micropterus salmoides* Kim et al. [29] reported a substantial rise in superoxide dismutase activity in the livers of juvenile hybrid groupers exposed to varied sublethal amounts of ammonia (1, 2, 4, and 8 mg/L). In addition, glutathione, an important cellular antioxidant, also scavenges ROS to protect cells from oxidative stress [30]. The glutathione levels in large yellow croaker livers increased considerably following ammonia exposure, indicating that the fish avoided the impacts of oxidative stress on the organism by increasing glutathione levels.

When free radicals are not completely scavenged by the antioxidant system, it leads to the accumulation of free radicals. Excess free radicals stimulate the production of malondialdehyde [15]. Malondialdehyde is a key product of lipid peroxidation and can reflect the extent of liver damage [31]. Malondialdehyde levels were significantly elevated in the livers of ammonia-treated samples, confirming the presence of oxidative stress in liver tissue and leading to membrane lipid peroxidation. The liver is a detoxification organ, and when the liver is damaged, blood levels of glutamate aminotransferase and glutamate pyruvic transaminase are elevated [32]. Therefore, elevated levels of glutamate pyruvic transaminase and glutamine transaminase indicate liver damage from acute ammonia exposure. The higher the ammonia level, the more severe the damage. Zhang et al. [23] also found the similar results that acute ammonia exposure generated a considerable rise in glutamic oxaloacetic transaminase and glutamic pyruvic transaminase, leading to liver damage in *Lateolabrax maculatus*. Lactate dehydrogenase is a key glycolytic enzyme that is produced when fish tissue is injured [33]. Continued exposure to ammonia can damage liver tissue and trigger elevated lactate dehydrogenase levels. Fish may increase their metabolic rate in response to high concentrations of ammonia, resulting in higher lactate dehydrogenase levels [22]. In addition, changes in lactate dehydrogenase levels were strongly correlated with ammonia levels and exposure time, suggesting that high levels of ammonia exposure accelerated metabolic rates and glycolysis, leading to increased lactate dehydrogenase levels. Protein and albumin produced by the liver are indicators of liver disease [34]. Ammonia leads to protein deamidation and damage to the kidneys and liver of fish, resulting in increased protein degradation and significant blood protein loss [35]. Serum total protein and albumin decreased significantly more than in CK when fish were exposed to ammonia. This may be attributed to the enhanced protease catalysis as well as protein hydrolysis activities that provide energy to the fish under sustained ammonia exposure [36].

Triglycerides and blood urea nitrogen are metabolites produced by the liver and kidneys during ammonia metabolism, and they are sensitive indicators for assessing renal injury and dysfunction. An increase in triglyceride and blood urea nitrogen contents in serum indicates that the kidney tissue had been damaged. The increase in plasma triglycerides in juvenile *Oncorhynchus mykiss* under acute ammonia stress was consistent with our result [37]. However, Esmaeili et al. [37] demonstrated that blood urea nitrogen in juvenile *Oncorhynchus mykiss* did not significantly change during acute ammonia stress. The differences in metabolic pathways and the ability of fish in converting ammonia to urea may be the reason for this result [38].

The gills are the primary organ for filtering and absorbing many harmful chemicals, including ammonia. The adaptation of gill shape to ammonia stress in the environment might represent a protective mechanism. The distance between the water and the gill vessels increases as the gill filaments thicken, reducing the pace at which harmful chemicals entered the circulation. Furthermore, the thickness reduced the respiratory surface area, making the large yellow croaker less vulnerable to hazardous chemicals like ammonia. In the response experiment of three teleost fishes to high ambient ammonia, Sinha et al. [39] discovered that exposure to ammonia led to substantial alterations in gill morphology.

Ammonia stress has been shown to promote the conversion of liver glycogen to glutamate, which provides the body with the energy it needs. The current study reports significantly elevated serum glucose levels in fish exposed to ammonia. This was attributed to the large amount of energy required to cope with persistent ammonia stress, which induced greater glucose production from hepatic glycogen metabolism. At the same time, higher ammonia exposure caused more severe stress in fish, which required more energy to cope with the stress. Gao et al. [8] found a similar result that significant increase in glucose contents in the serum of *Takifugu rubripes* with increasing ammonia exposure. In addition, ammonia disrupts lipid homeostasis in fish [40]. In the current study, the increased metabolic demand may contribute to the reduction in blood triglyceride levels under sustained ammonia exposure. Zhao et al. [41] also found that exposure to 2.65 mg/L ammonia significantly reduced serum triglyceride levels in *Pelteobagrus fulvidraco*.

Ammonia exposure has negative effects on fish, and as a result, fish have developed a variety of defense mechanisms against ammonia poisoning. One of the strategies is to convert excess ammonia in tissues into various nonessential FAAs. The current investigation found that increasing ammonia exposure content and exposure duration resulted in the accumulation of total FAAs, which was in line with Wang et al.’s [42] findings. In addition, fish that were not fed during the test period may have accelerated the degradation of fish proteins, leading to the accumulation of total FAAs. Glutamate and aspartate are key ammonia detoxification enzymes, and glutamate may react with ammonia to form glutamine in the presence of glutamine synthetase [43]. Thus, fish will respond to high ammonia environments by requiring large amounts of glutamate to convert ammonia into less toxic chemicals. ATP is predominant in live fish muscle but is rapidly degraded to IMP, AMP, and other related associates that affect the flavor of fish. The unique freshness of rhubarb fish is directly related to these flavor-presenting nucleotides [44]. The results showed that IMP contributes the most to the flavor characteristics of fish meat. ATP is rapidly degraded to IMP by endogenous enzymes in fish muscle, while IMP is degraded slowly [45]. Ammonia exposure accelerates the rate of flavor amino acid degradation, resulting in the loss of fish flavor.

## 5. Conclusions

The current research demonstrated that acute ammonia exposure caused considerable toxic effects in the large yellow croaker, including the induction of oxidative stress and liver tissue damage. Additionally, we found that the flavor nucleotides such as IMP and AMP were degraded by acute ammonia exposure. Exposure to TAN concentrations between 5.92 and 8.87 mg/L could increase the activities of catalase, glutamic oxaloacetic transaminase, lactate dehydrogenase, superoxide dismutase, and glutamic pyruvic transaminase; elevate the levels of glutathione, blood urea nitrogen, and malondialdehyde; and decrease the contents of total protein and albumin in large yellow croakers. The change degree was proportional to ammonia exposure content and exposure duration. Furthermore, during ammonia exposure, the thickening and shortening of gill lamellae were observed, especially in the NH-5.92 and NH-8.87 groups. Exposure to TAN concentrations between 5.92 and 8.87 mg/L also caused the accumulation of FAAs. Therefore, 5.92 and 8.87 mg/L TAN levels should be prevented in an applied scenario. Future research should look at the link between antioxidant enzyme expression and ammonia levels by analyzing the content of antioxidant enzyme-related genes in large yellow croakers. In addition, longer experiments should be conducted to verify that the change in gill morphology of large yellow croakers is to protect the body from ammonia toxicity. In conclusion, this work provides a foundation for deeper understanding of the ammonia mechanism process in the large yellow croaker.

## Figures and Tables

**Table 1:** Results of two-way ANOVA on the interaction between time or/and ammonia exposure on the levels of hematological biochemical parameters, and the activity of antioxidant enzyme-related indicators.

Parameters	Source of Variation	df	F	*p*
GOT	Time	6	753.743	<0.01
	Ammonia exposure	3	2533.762	<0.01
	Time × ammonia exposure	18	127.196	<0.01
GPT	Time	6	74.454	<0.01
	Ammonia exposure	3	138.426	<0.01
	Time × ammonia exposure	18	18.357	<0.01
LDH	Time	6	79.562	<0.01
	Ammonia exposure	3	171.74	<0.01
	Time × ammonia exposure	18	20.057	<0.01
SOD	Time	6	23,292.285	<0.01
	Ammonia exposure	3	30,863.541	<0.01
	Time × ammonia exposure	18	2749.248	<0.01
CAT	Time	6	113.854	<0.01
	Ammonia exposure	3	384.814	<0.01
	Time × ammonia exposure	18	24.854	<0.01
MDA	Time	6	171.04	<0.01
	Ammonia exposure	3	125.578	<0.01
	Time × ammonia exposure	18	14.12	<0.01
GSH	Time	6	83.025	<0.01
	Ammonia exposure	3	226.947	<0.001
	Time × ammonia exposure	18	20.986	<0.01
TP	Time	6	199.622	<0.01
	Ammonia exposure	3	221.744	<0.01
	Time × ammonia exposure	18	26.448	<0.01
Alb	Time	6	7.557	<0.01
	Ammonia exposure	3	9.586	<0.01
	Time × ammonia exposure	18	16.636	<0.01
Glu	Time	6	171.851	<0.01
	Ammonia exposure	3	50.009	<0.01
	Time × ammonia exposure	18	13.968	<0.01
TG	Time	6	443.666	<0.01
	Ammonia exposure	3	504.571	<0.01
	Time × ammonia exposure	18	38.161	<0.01
CRE	Time	6	141.983	<0.01
	Ammonia exposure	3	247.119	<0.01
	Time × ammonia exposure	18	20.672	<0.01
BUN	Time	6	101.202	<0.01
	Ammonia exposure	3	58.409	<0.01
	Time × ammonia exposure	18	4.765	<0.01

**Table 2:** Changes in free amino acids (FAAs) in the flesh of large yellow croakers exposed to 0 mg/L, 2.96 mg/L, 5.92 mg/L, and 8.87 mg/L total ammonia nitrogen for 48 h, respectively (CK, NH-2.96, NH-5.92, and NH-8.87 represent 0 mg/L, 2.96 mg/L, 5.92 mg/L, and 8.87 mg/L, respectively).

Samples	Time of Exposure (h)	Free Amino Acids							
		Aspartic Acid (Asp)	Threonine (Thr)	Serine (Ser)	Glutamic Acid (Glu)	Glycine (Gly)	Alanine (Ala)	Valine (Val)	Methionine (Met)
CK	0 h	0.88 ± 0.03 A	3.10 ± 0 A	3.00 ± 0 A	13.71 ± 0.01 A	6.78 ± 0.03 A	13.70 ± 0 A	8.59 ± 0.02 A	7.20 ± 0.01 A
	6 h	0.71 ± 0.01 C	2.61 ± 0.01 D	2.52 ± 0.03 D	12.11 ± 0.01 B	5.61 ± 0.01 D	11.39 ± 0.02 D	7.80 ± 0 D	6.80 ± 0 C
	12 h	3.91 ± 0.01 A	3.49 ± 0.02 D	4.00 ± 0.01 D	5.79 ± 0.02 D	7.41 ± 0.01 D	20.11 ± 0.01 B	5.28 ± 0.04 D	2.98 ± 0.03 D
	24 h	3.89 ± 0.01 B	3.42 ± 0.03 D	3.98 ± 0.03 D	5.72 ± 0.02 D	7.39 ± 0.02 C	20.00 ± 0.01 C	5.28 ± 0.03 D	2.98 ± 0.03 D
	36 h	0.91 ± 0.01 D	3.81 ± 0.01 C	3.51 ± 0.01 D	13.42 ± 0.03 C	6.40 ± 0.01 D	13.1 ± 0.01 D	9.58 ± 0.03 C	7.69 ± 0.01 C
	42 h	2.91 ± 0.01 B	4.42 ± 0.03 D	4.80 ± 0 D	7.51 ± 0.01 D	9.03 ± 0.04 D	19.42 ± 0.03 D	6.51 ± 0.01 C	3.90 ± 0 C
	48 h	2.91 ± 0.01 D	4.48 ± 0.03 D	4.81 ± 0.01 D	7.50 ± 0.01 D	9.08 ± 0.04 D	19.71 ± 0.01 D	6.48 ± 0.04 C	3.90 ± 0.01 C
NH-2.96	0 h	0.88 ± 0.03 A	3.10 ± 0 A	3.00 ± 0 A	13.71 ± 0.01 A	6.78 ± 0.03 A	13.70 ± 0 A	8.59 ± 0.02 A	7.20 ± 0.01 A
	6 h	1.72 ± 0.02 B	5.9 ± 0.01 A	4.80 ± 0.01 B	10.18 ± 0.03 D	8.29 ± 0.02 B	19.50 ± 0 B	8.59 ± 0.01 C	4.81 ± 0.01 D
	12 h	1.79 ± 0.02 C	6.00 ± 0 B	4.82 ± 0.02 B	10.42 ± 0.02 C	8.32 ± 0.03 B	19.89 ± 0.02 C	8.63 ± 0.04 C	4.90 ± 0 C
	24 h	2.19 ± 0.02 D	5.28 ± 0.04 A	5.83 ± 0.04 A	8.19 ± 0.02 C	15.41 ± 0.01 A	28.21 ± 0.01 A	8.71 ± 0.01 B	4.92 ± 0.02 B
	36 h	2.12 ± 0.03 C	5.20 ± 0 A	5.72 ± 0.03 A	8.02 ± 0.03 D	15.31 ± 0.01 A	28.02 ± 0.02 A	8.68 ± 0.03 D	4.89 ± 0.02 D
	42 h	5.29 ± 0.02 A	5.09 ± 0.01 C	6.90 ± 0 B	10.61 ± 0.01 C	12.90 ± 0.01 B	25.62 ± 0.02 B	5.59 ± 0.01 D	3.09 ± 0.02 D
	48 h	5.40 ± 0 A	5.21 ± 0.01 B	7.00 ± 0 B	10.93 ± 0.04 C	13.12 ± 0.03 B	26.21 ± 0.01 B	5.80 ± 0 D	3.21 ± 0.01 D
NH-5.92	0 h	0.88 ± 0.03 A	3.10 ± 0 A	3.00 ± 0 A	13.71 ± 0.01 A	6.78 ± 0.03 A	13.70 ± 0 A	8.59 ± 0.02 A	7.20 ± 0.01 A
	6 h	2.50 ± 0.01 A	3.32 ± 0.03 C	3.31 ± 0.01 C	18.81 ± 0.01 A	9.42 ± 0.03 A	12.92 ± 0.03 C	9.71 ± 0.01 B	7.22 ± 0.03 B
	12 h	2.62 ± 0.02 B	7.49 ± 0.02 A	6.88 ± 0.04 A	28.08 ± 0.04 A	14.92 ± 0.03 A	17.99 ± 0.01 D	11.99 ± 0.02 B	8.89 ± 0.01 A
	24 h	4.58 ± 0.03 A	5.31 ± 0.01 A	5.71 ± 0.01 B	12.12 ± 0.02 B	10.59 ± 0.01 B	20.99 ± 0.02 B	8.70 ± 0.01 C	4.51 ± 0.01 C
	36 h	3.89 ± 0.02 A	5.19 ± 0.01 A	5.20 ± 0 B	28.83 ± 0.04 A	14.98 ± 0.04 B	20.59 ± 0.01 B	13.00 ± 0 B	8.48 ± 0.03 B
	42 h	2.50 ± 0.01 C	7.11 ± 0.01 B	10.69 ± 0.01 A	28.01 ± 0.01 A	26.21 ± 0.01 A	22.4 ± 0.01 C	11.8 ± 0 B	8.41 ± 0.01 B
	48 h	3.12 ± 0.03 C	9.08 ± 0.03 A	8.20 ± 0.01 A	33.82 ± 0.02 A	18.29 ± 0.02 A	21.91 ± 0.01 C	13.8 ± 0.01 B	9.81 ± 0.01 A
NH-8.87	0 h	0.88 ± 0.03 A	3.10 ± 0 A	3.00 ± 0 A	13.71 ± 0.01 A	6.78 ± 0.03 A	13.70 ± 0 A	8.59 ± 0.02 A	7.20 ± 0.01 A
	6 h	1.73 ± 0.04 B	4.72 ± 0.03 D	4.89 ± 0.02 A	11.09 ± 0.02 C	8.10 ± 0 C	30.88 ± 0.04 A	12.31 ± 0.01 A	8.21 ± 0.01 A
	12 h	1.80 ± 0.01 C	5.51 ± 0.01 C	4.70 ± 0 C	11.60 ± 0 B	7.49 ± 0.02 C	29.80 ± 0.01 A	13.20 ± 0.01 A	7.79 ± 0.01 B
	24 h	2.59 ± 0.01 C	4.78 ± 0.03 B	4.33 ± 0.04 C	26.81 ± 0.01 A	6.58 ± 0.03 D	19.62 ± 0.02 D	13.31 ± 0.01 A	8.52 ± 0.02 A
	36 h	2.60 ± 0.01 B	4.80 ± 0.01 B	4.39 ± 0.01 C	26.92 ± 0.03 B	6.62 ± 0.02 C	19.69 ± 0.01 C	13.31 ± 0.01 A	8.62 ± 0.02 A
	42 h	2.48 ± 0.03 C	7.50 ± 0 A	6.12 ± 0.03 C	15.92 ± 0.03 B	10.40 ± 0 C	41.20 ± 0 A	16.18 ± 0.04 A	8.91 ± 0.01 A
	48 h	5.29 ± 0.01 B	5.11 ± 0.01 C	5.02 ± 0.02 C	26.80 ± 0.01 B	9.41 ± 0.01 C	26.79 ± 0.02 A	15.30 ± 0.01 A	9.39 ± 0.02 B
**Samples**	**Time of Exposure (h)**	**Free Amino Acids**							
		**Isoleucine (Ile)**	**Leucine (Leu)**	**Tyrosine (Tyr)**	**Phenylalanine (Phe)**	**Lysine (Lys)**	**NH3**	**Histidine (His)**	**Total**
CK	0 h	5.15 ± 0.21 A	7.79 ± 0.3 A	2.99 ± 0.02 A	4.84 ± 0.23 A	19.84 ± 0.23 A	9.99 ± 0.02 A	4.92 ± 0.12 A	112.46
	6 h	4.86 ± 0.21 C	6.24 ± 0.34 C	2.81 ± 0.27 D	4.15 ± 0.21 C	17.12 ± 0.17 A	8.07 ± 0.09 D	4.76 ± 0.34 B	97.53
	12 h	2.82 ± 0.26 D	7.24 ± 0.33 D	6.93 ± 0.11 B	28.03 ± 0.04 A	14.97 ± 0.04 B	17.99 ± 0.01 B	11.99 ± 0.02 A	142.88
	24 h	3.98 ± 0.03 C	6.22 ± 0.3 C	2.94 ± 0.09 C	3.04 ± 0.05 C	7.85 ± 0.21 D	21.12 ± 0.17 B	2.80 ± 0.29 D	100.58
	36 h	6.23 ± 0.33 C	9.25 ± 0.35 D	4.06 ± 0.08 C	5.80 ± 0.29 C	11.06 ± 0.08 C	10.19 ± 0.26 D	4.76 ± 0.34 B	109.73
	42 h	4.95 ± 0.07 C	8.12 ± 0.16 C	3.92 ± 0.12 B	4.15 ± 0.21 C	11.08 ± 0.11 D	19.97 ± 0.04 B	3.04 ± 0.05 C	113.69
	48 h	4.95 ± 0.08 C	8.11 ± 0.16 C	3.94 ± 0.08 C	4.13 ± 0.18 C	11.01 ± 0.01 D	20.1 ± 0.14 B	3.01 ± 0.01 D	114.08
NH-2.96	0 h	5.15 ± 0.21 A	7.79 ± 0.3 A	2.99 ± 0.02 A	4.84 ± 0.23 A	19.84 ± 0.23 A	9.99 ± 0.02 A	4.92 ± 0.12 A	112.46
	6 h	6.91 ± 0.13 B	10.14 ± 0.19 B	4.23 ± 0.32 B	4.96 ± 0.06 B	14.14 ± 0.2 B	21.24 ± 0.34 A	3.00 ± 0.01 D	128.38
	12 h	6.92 ± 0.12 C	10.76 ± 0.34 C	4.81 ± 0.28 C	5.05 ± 0.06 C	14.82 ± 0.25 B	21.25 ± 0.35 A	3.03 ± 0.04 D	131.36
	24 h	6.95 ± 0.08 B	10.24 ± 0.34 B	4.88 ± 0.18 B	5.18 ± 0.25 B	9.85 ± 0.21 C	19.96 ± 0.06 C	3.80 ± 0.29 B	139.55
	36 h	6.95 ± 0.07 C	10.11 ± 0.16 C	4.81 ± 0.28 B	5.04 ± 0.06 D	9.23 ± 0.33 D	19.96 ± 0.06 A	3.23 ± 0.32 C	137.26
	42 h	3.98 ± 0.03 D	6.78 ± 0.31 D	3.18 ± 0.25 C	3.90 ± 0.14 D	30.04 ± 0.06 A	24.75 ± 0.35 A	3.85 ± 0.21 B	151.55
	48 h	4.05 ± 0.07 D	6.87 ± 0.18 D	3.23 ± 0.33 D	3.87 ± 0.19 D	31.07 ± 0.09 A	24.92 ± 0.11 A	3.96 ± 0.06 C	154.82
NH-5.92	0 h	5.15 ± 0.21 A	7.79 ± 0.3 A	2.99 ± 0.02 A	4.84 ± 0.23 A	19.84 ± 0.23 A	9.99 ± 0.02 A	4.92 ± 0.12 A	112.46
	6 h	6.24 ± 0.34 B	10.06 ± 0.08 B	3.23 ± 0.33 C	4.87 ± 0.19 B	13.12 ± 0.16 C	10.02 ± 0.03 B	4.17 ± 0.23 C	118.90
	12 h	8.89 ± 0.16 B	13.81 ± 0.28 B	4.00 ± 0.01 D	5.80 ± 0.29 C	16.99 ± 0.02 A	12.94 ± 0.09 C	5.99 ± 0.02 B	167.22
	24 h	6.91 ± 0.13 B	10.23 ± 0.32 B	4.77 ± 0.33 B	5.22 ± 0.31 B	16.20 ± 0.28 B	22.22 ± 0.31 A	3.18 ± 0.25 C	141.21
	36 h	9.17 ± 0.23 B	15.21 ± 0.29 B	5.01 ± 0.01 B	6.08 ± 0.11 B	18.98 ± 0.03 B	14.77 ± 0.33 B	5.18 ± 0.25 A	174.54
	42 h	8.15 ± 0.21 B	12.94 ± 0.08 B	3.99 ± 0.01 B	5.80 ± 0.29 B	15.22 ± 0.31 B	13.23 ± 0.32 C	6.97 ± 0.05 A	183.38
	48 h	10.20 ± 0.28 B	16.21 ± 0.29 B	4.91 ± 0.13 B	6.16 ± 0.22 B	19.87 ± 0.18 C	14.93 ± 0.11 D	6.80 ± 0.28 A	197.05
NH-8.87	0 h	5.15 ± 0.21 A	7.79 ± 0.3 A	2.99 ± 0.02 A	4.84 ± 0.23 A	19.84 ± 0.23 A	9.99 ± 0.02 A	4.92 ± 0.12 A	112.46
	6 h	8.81 ± 0.27 A	14.02 ± 0.03 A	5.23 ± 0.32 A	6.11 ± 0.16 A	14.00 ± 0.01 B	8.92 ± 0.11 C	5.80 ± 0.28 A	144.79
	12 h	9.98 ± 0.03 A	14.91 ± 0.13 A	7.02 ± 0.03 A	7.22 ± 0.31 B	9.82 ± 0.25 C	10.07 ± 0.09 D	5.23 ± 0.32 C	146.10
	24 h	10.00 ± 0.01 A	16.08 ± 0.11 A	6.13 ± 0.18 A	6.76 ± 0.35 A	24.14 ± 0.2 A	11.95 ± 0.08 D	5.11 ± 0.15 A	166.65
	36 h	10.08 ± 0.11 A	16.05 ± 0.06 A	6.15 ± 0.21 A	6.79 ± 0.3 A	24.82 ± 0.25 A	11.14 ± 0.19 C	5.14 ± 0.2 A	167.08
	42 h	12.84 ± 0.23 A	19.17 ± 0.24 A	8.99 ± 0.02 A	9.06 ± 0.08 A	12.24 ± 0.34 C	13.10 ± 0.13 D	6.78 ± 0.32 A	190.87
	48 h	12.07 ± 0.09 A	19.92 ± 0.11 A	7.16 ± 0.22 A	7.77 ± 0.33 A	23.87 ± 0.18 B	16.18 ± 0.25 C	5.25 ± 0.35 B	195.28

Different capital letters indicate significant differences in means between different treatment samples (*p* < 0.05).

**Table 3:** Changes in nucleotides of large yellow croakers exposed to 0 mg/L, 2.96 mg/L, 5.92 mg/L, and 8.87 mg/L total ammonia nitrogen for 48 h, respectively (CK, NH-2.96, NH-5.92, and NH-8.87 represent 0 mg/L, 2.96 mg/L, 5.92 mg/L, and 8.87 mg/L, respectively).

Samples	Time of Exposure (h)	Inosine Monophosphate (IMP) (mg/100 g)	TAV	Adenosine Monophosphate (AMP) (mg/100 g)	TAV
CK	0 h	273.76 ± 0.01 Aa	10.95	19.42 ± 0.59 Aa	0.40
	6 h	264.1 ± 0.01 Aab	10.56	17.1 ± 0.13 Ab	0.34
	12 h	264.07 ± 0.01 Aab	10.56	16.02 ± 0.02 Ac	0.32
	24 h	263.92 ± 0.01 Aab	10.56	15.15 ± 0.21 Ad	0.31
	36 h	262.03 ± 0.01 Aab	10.48	15.06 ± 0.08 Ad	0.30
	42 h	259.84 ± 0.01 Ab	10.39	14.06 ± 0.08 Ae	0.28
	48 h	253.58 ± 0.01 Ab	10.14	13.35 ± 0.49 Ae	0.27
NH-2.96	0 h	273.76 ± 0.01 Aa	9.28	19.42 ± 0.59 Aa	0.31
	6 h	231.88 ± 0.01 Cb	9.54	15.24 ± 0.34 Bb	0.28
	12 h	238.53 ± 0.01 Bc	8.57	14.04 ± 0.06 Bc	0.27
	24 h	214.31 ± 0.01 Bd	6.04	13.25 ± 0.35 Bd	0.25
	36 h	150.96 ± 0.01 Be	5.80	12.13 ± 0.18 Be	0.23
	42 h	145.07 ± 0.01 Be	5.96	11.14 ± 0.2 Bf	0.19
	48 h	148.91 ± 0.01 Bfe	5.96	9.24 ± 0.34 Bg	0.19
NH-5.92	0 h	273.76 ± 0.01 Aa	10.95	19.42 ± 0.59 Aa	0.40
	6 h	256.04 ± 0.03 Bb	10.24	14.38 ± 0.53 BCb	0.30
	12 h	198.14 ± 0.12 Cc	7.93	9.35 ± 0.49 Cd	0.19
	24 h	178.17 ± 0.15 Cd	7.13	11.47 ± 0.66 Cc	0.24
	36 h	131.19 ± 0.17 Cf	5.25	9.25 ± 0.35 Cd	0.19
	42 h	167.18 ± 0.16 Ce	6.69	8.01 ± 0.01 Ce	0.16
	48 h	142.03 ± 0.03 Cg	5.68	9 ± 0 dBe	0.18
NH-8.87	0 h	273.51 ± 0.44 Aa	10.95	19.42 ± 0.59 Aa	0.40
	6 h	199.32 ± 0.28 Db	7.98	14.2 ± 0.28 Cb	0.29
	12 h	141.17 ± 0.15 Dc	5.65	12.48 ± 0.67 Dc	0.26
	24 h	133.31 ± 0.27 Dd	5.34	11.1 ± 0.14 Cd	0.22
	36 h	117.33 ± 0.28 De	4.70	9.12 ± 0.17 Ce	0.18
	42 h	107.25 ± 0.22 Df	4.30	6.17 ± 0.23 Df	0.13
	48 h	99.53 ± 0.46 Df	3.99	6.1 ± 0.14 Cf	0.12

Different capital letters indicate significant differences in means between different treatment groups. Different lowercase letters indicate significant differences in means between the same treatment group (*p* < 0.05).

## Data Availability

The data presented in this study are available upon request from the corresponding author.
